# Distinct Transmission Networks of *Chlamydia trachomatis* in Men Who Have Sex with Men and Heterosexual Adults in Amsterdam, The Netherlands

**DOI:** 10.1371/journal.pone.0053869

**Published:** 2013-01-16

**Authors:** Reinier J. M. Bom, Jannie J. van der Helm, Maarten F. Schim van der Loeff, Martijn S. van Rooijen, Titia Heijman, Amy Matser, Henry J. C. de Vries, Sylvia M. Bruisten

**Affiliations:** 1 Public Health Laboratory, Public Health Service of Amsterdam (GGD Amsterdam), Amsterdam, The Netherlands; 2 Department of Research, Public Health Service of Amsterdam (GGD Amsterdam), Amsterdam, The Netherlands; 3 STI Outpatient Clinic, Public Health Service of Amsterdam (GGD Amsterdam), Amsterdam, The Netherlands; 4 Center for Infection and Immunology Amsterdam (CINIMA), Academic Medical Center (AMC), University of Amsterdam, Amsterdam, The Netherlands; 5 Julius Centre for Health Sciences & Primary Care, University Medical Centre Utrecht (UMCU), Utrecht, The Netherlands; 6 Department of Dermatology, Academic Medical Center (AMC), University of Amsterdam, Amsterdam, The Netherlands; University of California, San Francisco, University of California Berkeley, and the Children's Hospital Oakland Research Institute, United States of America

## Abstract

**Background:**

Genovar distributions of *Chlamydia trachomatis* based on *ompA* typing differ between men who have sex with men (MSM) and heterosexuals. We investigated clonal relationships using a high resolution typing method to characterize *C. trachomatis* types in these two risk groups.

**Methods:**

*C. trachomatis* positive samples were collected at the STI outpatient clinic in Amsterdam between 2008 and 2010 and genotyped by multilocus sequence typing. Clusters were assigned using minimum spanning trees and these were combined with epidemiological data of the hosts.

**Results:**

We typed 526 *C. trachomatis* positive samples: 270 from MSM and 256 from heterosexuals. Eight clusters, containing 10–128 samples were identified of which 4 consisted of samples from MSM (90%–100%), with genovars D, G, J, and L2b. The other 4 clusters consisted mainly of samples from heterosexuals (87%–100%) with genovars D, E, F, I, and J. Genetic diversity was much lower in the MSM clusters than in heterosexual clusters. Significant differences in number of sexual partners and HIV-serostatus were observed for MSM–associated clusters.

**Conclusions:**

*C. trachomatis* transmission patterns among MSM and heterosexuals were largely distinct. We hypothesize that these differences are due to sexual host behavior, but bacterial factors may play a role as well.

## Introduction

*Chlamydia trachomatis* propagates as an obligate intracellular pathogen that is easily transmitted sexually and often causes asymptomatic infections. *C. trachomatis* is endemic in the general population, but the majority of infections occur within transmission networks of specific risk groups, such as heterosexual adolescents, young adults, and men who have sex with men (MSM) [1]. Low resolution typing methods, based on the *ompA* gene, showed that multiple genovars circulate. Three genovars (D, E, and F) comprise about 70% of the infections among heterosexuals, and the distribution of genovars appears to be stable over time and independent of clinical symptoms and geography [2]–[6]. The distribution among MSM appears to be different; genovars D, G, and J comprise about 85% of *C. trachomatis* infections among MSM according to a limited number of studies [5], [7]. Recently, a fourth dominant genovar, L2b, was added to this distribution, because a lymphogranuloma venereum (LGV) outbreak occurred among MSM in Europe, North America, and Australia [8]–[10].

In this study, differences in circulating *C. trachomatis* strains between MSM and heterosexuals were investigated using a high resolution typing method. A modified multilocus sequence typing (MLST) technique was used, which was epidemiologically validated on clinical samples to differentiate *C. trachomatis* strains on a population level [11]. Using this technique, we previously found that there were differences in genetic diversity among *C. trachomatis* strains from MSM and heterosexual women sampled in 3 different countries [12]. These hosts were separated in calendar time and geography however, making it unlikely that direct exchange of Chlamydia strains had been possible. In the present study, we focused on only one STI clinic in one city, Amsterdam, the Netherlands. Samples were collected from both MSM and heterosexual men and women in a relatively short time frame (2008–2010). We aimed to investigate the diversity of chlamydial genotypes, and analyzed epidemiological characteristics of *C. trachomatis* MLST clusters between the risk groups.

## Materials and Methods

### Study Site

The study was conducted among visitors of the STI outpatient clinic of the Public Health Service of Amsterdam, the Netherlands. The low-threshold clinic offers free-of-charge testing and treatment for STI to more than 30,000 visitors a year, of whom 29% are MSM [13]. At entry, visitors are classified as high or low risk depending on reported sexual behavior. Criteria for high-risk classification were: having STI-related physical complaints; being notified of STI exposure by a sexual partner; having been paid for sexual contact in the past 6 months; and for males, having had sex with men in the past 6 months. The high- and low-risk groups are assigned to standard and short screening protocols, respectively. An earlier study had shown that these criteria were able to identify groups with a high prevalence of STI. Both protocols include diagnostic testing for *C. trachomatis* infections [13]. Urine and swabs (taken from the vagina, cervix, urethra, proctum, or ulcer, depending on sexual history and clinical symptoms) were tested for *C. trachomatis* upon collection, using transcription-mediated amplification (TMA; Aptima Combo 2, Gen-Probe, San Diego, USA) at the Public Health Laboratory.

### Study Population

For logistic reasons, recruitment for the 2 risk groups could not be conducted concurrently. Therefore recruitment was done consecutively for each group with the collection periods separated by two months. In addition, participants were only recruited from the standard screening protocol. The first recruited group consisted of MSM at risk for STI. Inclusion criteria were being at least 18 years old, understanding of written Dutch or English, and having had sex with a man in the preceding 6 months. The recruitment period ran from July 2008 through August 2009. The second group consisted of male heterosexuals and females at risk for STI. Male visitors were excluded if they had had sex with a male partner in the preceding 6 months. Visitors had to understand written Dutch or English and be at least 18 years old. The recruitment period ran from November 2009 through May 2010. Visitors could participate only once in this study, with one sample. For MSM, urine samples were used or, if urine samples were not available, proctal samples were used. For heterosexual men, only urine samples were used. For female participants, vaginal self-swab samples were used or, if not available, cervical samples were used. Epidemiological data, such as diagnosis at current visit and medical and sexual history, were obtained from the electronic patient record. The study was approved by the medical ethics committee of the Academic Medical Centre of Amsterdam, the Netherlands (MEC07/127, MEC07/181). Participants gave written informed consent.

### DNA Extraction

DNA was extracted from clinical samples using 200 µL out of the 4 mL of transport medium (Gen-Probe), containing swabs or urine that had previously been tested positive for *Chlamydia trachomatis* by routine analysis (Aptima Combo 2, Gen-Probe, San Diego, USA). The medium was added to 500 µL lysis buffer (bioMérieux, Boxtel, the Netherlands), 1 µL glycogen (20 mg/ml, Roche Diagnostics, Almere, the Netherlands). After mixing and incubation at 65°C for 30 minutes, 700 µL ice-cold isopropanol was added. The precipitate was centrifuged for 20 minutes at 14000 rpm, washed twice with 70% ethanol, and dissolved in 50 µL 10 mM Tris buffer (pH 8.0). DNA isolates were stored at −20°C until use. Per PCR reaction, 2 µL of DNA isolate was used.

### DNA Quantification

All DNA isolates were further tested with the in-house *pmpH* LGV qPCR for presence of genomic DNA as described previously [7], [14]. For isolates that were negative or obtained a cycling threshold (Ct) value higher than 35, DNA was re-extracted from the original samples and requantified. For samples that were retested, the DNA isolate with the lowest cycling threshold was selected for further typing analyses. Samples that tested repeatedly negative or weakly positive (Ct>40) were excluded from typing analysis.

### Nested PCR and Sequencing of MLST Regions

DNA isolates were amplified by a nested PCR for the regions *ompA*, CT046 (*hctB*), CT058, CT144, CT172, and CT682 (*pbpB*) as described previously [11]. There were some minor adaptations to increase sensitivity: the primers CT1678R and pbpB2366R were replaced by CT058IR (5′AAT CCT CCT TGG CCT CTC TT) and pbpB1OR (5′AAG AAC CTT CCA TCT CCT GAA T). The inner PCR was performed with M13-tagged primers, identical to the standard inner primers. This allowed sequencing using universal M13 primers for high throughput purposes.

### Data Analysis

The obtained sequences were analyzed, assembled, and trimmed from their primer sequences, using BioNumerics 6.6 (Applied Maths, Sint-Martens-Latem, Belgium). Samples resulting in incomplete and low-quality sequences were reamplified and resequenced. The cleaned primer-to-primer sequences were checked against an in-house library and against the *Chlamydia trachomatis* MLST database (mlstdb.bmc.uu.se); each different sequence from a region was given an allele number. Only samples of which all six alleles were successfully amplified, sequenced, and identified, were included in the study and obtained a full MLST profile. Samples with an incomplete MLST profile after repeated testing and samples containing double infections were excluded from further analysis. A minimum spanning tree was generated using MLST profiles. Cluster analysis was performed, allowing single locus variance (SLV), using BioNumerics 6.6. Clusters containing 10 or more samples were defined as large clusters. To investigate specific characteristics of small clusters (n≤9) and singletons, we combined these samples into a residual group.

### Statistical Analysis

Participants were classified into 2 groups, MSM and heterosexuals, on the basis of their sexual behavior. MSM were defined as those men who reported having sexual contact with men in the preceding 6 months. The group of MSM was subdivided into men who have sex with men only (MSMO) and men who have sex with both men and women (MSMW, bisexuals). Men reporting sexual contact with women only (MSWO) in the preceding 6 months were regarded as heterosexuals. In addition, all women were regarded as heterosexuals, as male-to-female transmission is most likely for *C. trachomatis* infections among women. Ethnicity was derived from the nationality, country of birth, and self-perceived ethnicity of the participants. If all categories were answered Dutch/the Netherlands, participants were classified as ‘Dutch’. If not, they were classified as ‘Other’. Differences between the 2 groups and between clusters were tested univariately using the Pearson’s *χ* ^2^ test, Fisher’s exact test, Mann-Whitney *U* test, and Kruskal-Wallis test, when appropriate. A *p*-value of ≤0.05 was considered statistically significant. Analyses were performed with SPSS package version 19.0 (SPSS Inc., Chicago, IL, USA).

The diversity within each clusters was calculated using Simpson’s index of diversity (*D*). The formula used to define this index is:
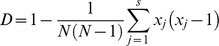
where *N* is the total number of samples, and *x_j_* the number of samples belonging to the *j* ^th^ sequence type and *s* is the number of different sequence types. Confidence intervals (95%*CI*) were estimated using:



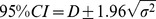
where the standard deviation *σ* is given by:



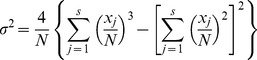



## Results

### Study Populations and Samples

We enrolled 3992 participants of the STI outpatient clinic in Amsterdam. Of these, 2492 were MSM and 1500 were heterosexuals. In total, 669 participants were positive for *C. trachomatis* by routine TMA testing (365 MSM and 304 heterosexuals). From these 669 participants, 658 *C. trachomatis* positive samples were available. In 566 samples (86%), the genomic DNA load was sufficiently high for genotyping (for 43 samples, the second extraction was selected). Of these samples, 526 (93%) could be completely typed by MLST (of which 30 samples came from the second extraction).

Among the 526 participants with a full MLST profile were 270 MSM and 256 heterosexuals (Table 1). Notable differences between the 2 groups were the higher age among MSM (*p*<0.001), higher reported number of sexual partners among MSM (*p*<0.001), and MSM being less often notified for an STI by sexual partners (*p*<0.001). Almost half of the MSM were HIV positive, versus none of the heterosexuals (*p*<0.001, Table 1). Coinfections with *Neisseria gonorrhoeae* were significantly more common among MSM (*p*<0.001, Table 1).

**Table 1:** Demographic and clinical characteristics of MSM and heterosexuals with Chlamydia trachomatis infection, STI outpatient clinic, Amsterdam, July 2008–May 2010.

		MSM (n = 270)		Heterosexuals (n = 256)		*p*
		n	(%)	n	(%)	
**Sex group**	*MSMO*	262	(97)	–	–	<0.001
	*MSMW*	8	(3)	–	–	
	*MSWO*	–	–	86	(34)	
	*Female*	–	–	170	(66)	
**Age in years**	*Median (IQR)*	39	(31–45)	23	(21–27)	<0.001
**Ethnicity** a	*Dutch*	191	(72)	183	(71)	0.935
	*Other*	75	(28)	73	(29)	
**Residence** b	*Amsterdam*	210	(80)	186	(75)	0.161
	*Outside Amsterdam*	51	(20)	61	(25)	
**Number of sexual partners in past 6 months** c	*Median (IQR)*	8	(4–18)	2	(1–4)	<0.001
**Notified for STI**	*No*	221	(82)	163	(64)	<0.001
	*Yes*	49	(18)	93	(36)	
**Source of sample**	*Urethra (male)*	89	(33)	86	(34)	<0.001
	*Proctum (male)*	181	(67)	–	–	
	*Cervix/Vagina*	–	–	170	(66)	
**STI-related complaints**	*No*	121	(45)	131	(51)	0.145
	*Yes*	149	(55)	125	(49)	
**HIV** d	*Negative*	144	(55)	254	(100)	<0.001
	*Positive*	116	(45)	0		
***Neisseria gonorrhoeae***	*Negative*	218	(81)	242	(95)	<0.001
	*Positive*	52	(19)	14	(5)	

MSM: men who have sex with men; MSMO: men who have sex with men only; MSMW: men who have sex with men and women; MSWO: men who have sex with women only; STI: sexually transmitted infections; IQR: interquartile range.

a Data were missing for 4 MSM.

b Data were missing for 9 MSM and 9 heterosexuals.

c Data were missing for 6 MSM.

d Data were missing for 10 MSM and 2 heterosexuals.

### Genovar Distributions

As *ompA* is part of the MLST scheme, genovars could be assigned to all typed samples. Among MSM, we found 7 different genovars: D (32%), E (4%), F (3%), G (32%), J (16%), K (0.4%), and L2b (13%). Among heterosexuals, we found 9 different genovars: B (1%), D (11%), E (39%), F (20%), G (6%), H (1%), I (12%), J (5%), and K (4%). Notable was the high prevalence of LGV genovar L2b among MSM and genovar I among heterosexuals.

### High Resolution Typing Results

**Figure 1:**
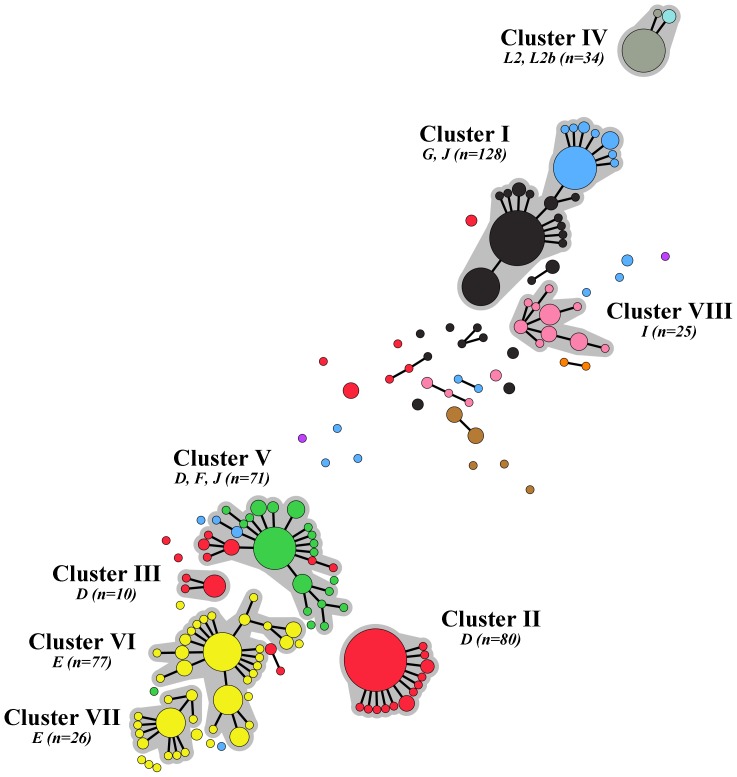
Minimum spanning tree showing *ompA* genovar type of 526 *Chlamydia trachomatis*-positive samples from MSM and heterosexuals in Amsterdam between July 2008 and May 2010. Sizes of the node discs are proportional to the number of samples of each sequence type; branches show single locus variants (SLV); halos indicate clusters based on SLV; and letters indicate *ompA* genovar type. The color coding is: red, genovar D (n = 115); yellow, genovar E (n = 112); black, genovar G (n = 102); green, genovar F (n = 61); blue, genovar J (n = 56); pink, genovar I (n = 31); gray, genovar L2b (n = 31); brown, genovar K (n = 11); cyan, genovar L2 (n = 3); purple, genovar B (n = 2); and orange, genovar H (n = 2).

Among the 526 fully typed samples, 164 unique sequence types were found (Table S1). Using the MLST profiles, a minimum spanning tree was generated, in which 8 large clusters could be identified (Figure 1). These clusters ranged from 10 to 128 samples and comprised 86% of all samples. The remaining 75 samples were distributed over 28 singletons and 16 small clusters, ranging from 2 to 8 samples.

**Figure 2:**
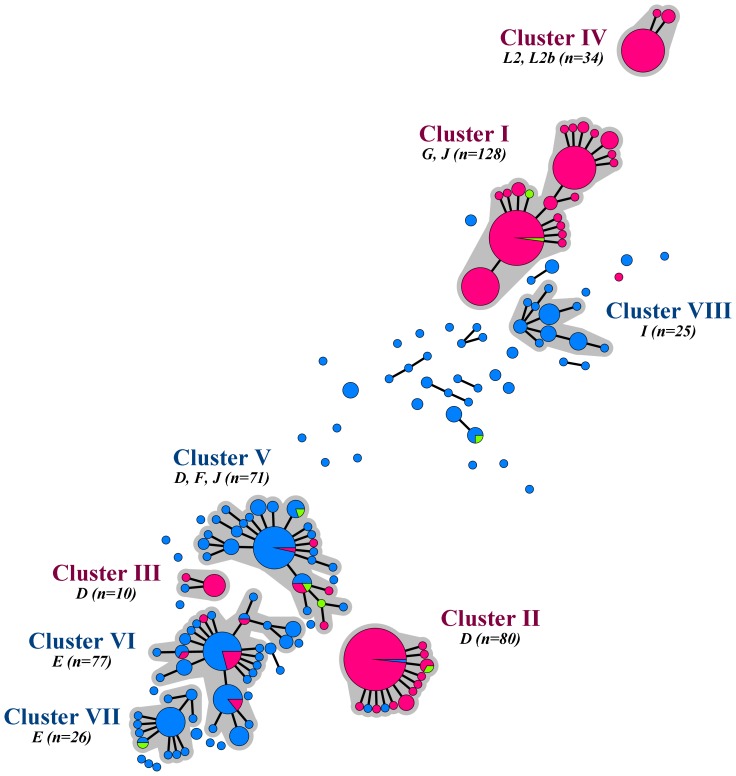
Minimum spanning tree showing the sexual orientation of the host of 526 *Chlamydia trachomatis*-positive samples from MSM and heterosexuals in Amsterdam between July 2008 and May 2010. Sizes of the node discs are proportional to the number of samples of each sequence type; branches show single locus variants (SLV); halos indicate clusters based on SLV; and letters indicate *ompA* genovar type. The color coding is: pink, men who have sex with men only (n = 262); green, men who have sex with men and women (n = 8); and blue, heterosexual men and women (n = 256).

A clear separation in the minimum spanning tree was seen between samples from MSM and heterosexuals (Figure 2). Four large clusters (clusters I to IV) consisted predominantly of samples from MSM (90% to 100%) and were therefore named MSM-associated clusters. The other 4 large clusters (clusters V to VIII) consisted primarily of samples from heterosexuals (87% to 100%) and were named heterosexual-associated clusters. Of the samples from 75 participants outside the large clusters, only 2 were from MSM.

Within the 4 MSM-associated clusters we identified genovars D, G, J, L2 and L2b (Table 2). Two clusters fully consisted of samples with an identical genovar: clusters II and III contained only genovar D samples. Cluster IV contained genovar L2b (91%) and L2 (9%) samples. Cluster I however consisted of genovar G samples (67%) as well as genovar J samples (33%). The same phenomenon was seen for the heterosexual-associated clusters (Table 2). Cluster V contained genovars F (82%), D (14%), and J (4%), while clusters VI and VII consisted of genovar E samples and cluster VIII of genovar I samples. The remaining small clusters and singletons contained a wide variety of genovars, with genovars G, D, J, and K being the most prominent.

**Table 2:** Demographic and clinical characteristics of participants by cluster based on MLST of Chlamydia trachomatis, STI outpatient clinic, Amsterdam, July 2008–May 2010.

		MSM-associated clusters				Heterosexual-associated clusters							
		Cluster I(n = 128)	Cluster II(n = 80)	Cluster III(n = 10)	Cluster IV(n = 34)	Cluster V(n = 71)	Cluster VI(n = 77)	Cluster VII(n = 26)	Cluster VIII(n = 25)	Residual Group(n = 75)	*p_total_*	*p_I to IV_*	*p_V to VIII+R_*
		n (%)	n (%)	n (%)	n (%)	n (%)	n (%)	n (%)	n (%)	n (%)			
***ompA*** ** genovar**	*B*	0	0	0	0	0	0	0	0	2 (3)	–	–	–
	*D*	0	80 (100)	10 (100)	0	10 (14)	0	0	0	15 (20)			
	*E*	0	0	0	0	0	77 (100)	26 (100)	0	9 (12)			
	*F*	0	0	0	0	58 (82)	0	0	0	3 (4)			
	*G*	86 (67)	0	0	0	0	0	0	0	16 (21)			
	*H*	0	0	0	0	0	0	0	0	2 (3)			
	*I*	0	0	0	0	0	0	0	25 (100)	6 (8)			
	*J*	42 (33)	0	0	0	3 (4)	0	0	0	11 (15)			
	*K*	0	0	0	0	0	0	0	0	11 (15)			
	*L2*	0	0	0	3 (9)	0	0	0	0	0			
	*L2b*	0	0	0	31 (91)	0	0	0	0	0			
**Simpson’s diversity index**	*D (95% CI)*	0.77 (0.72–0.82)	0. 38 (0. 24–0. 52)	0.38 (0.03–0.72)	0.22 (0.04–0.39)	0.82 (0.74–0.91)	0.86 (0.81–0.92)	0.71 (0.53–0.90)	0.87 (0.80–0.94)	–	–	–	–
**Sex group**	*MSMO*	126 (98)	76 (95)	9 (90)	34 (100)	6 (8)	10 (13)	0	0	1 (1)	<0.001	0.062	0.022
	*MSMW*	2 (2)	1 (1)	0	0	3 (4)	0	1 (4)	0	1 (1)			
	*MSWO*	0	3 (4)	0	0	16 (23)	29 (38)	7 (27)	9 (36)	22 (29)			
	*Female*	0	0	1 (10)	0	46 (65)	38 (49)	18 (69)	16 (64)	51 (68)			
**Age in years**	*Median (IQR)*	39 (31–45)	39 (29–45)	33 (24–42)	40 (35–45)	24 (21–27)	24 (21–28)	25 (22–28)	24 (21–27)	23 (21–32)	<0.001	0.126	0.708
**Ethnicity** a	*Dutch*	90 (71)	57 (71)	8 (89)	26 (79)	49 (69)	58 (76)	17 (65)	12 (48)	57 (76)	0.228	0.627	0.065
	*Other*	37 (29)	23 (29)	1 (11)	7 (21)	22 (31)	18 (24)	9 (35)	13 (52)	18 (24)			
**Residence** b	*Amsterdam*	99 (79)	64 (85)	9 (90)	24 (75)	56 (81)	51 (69)	18 (69)	16 (67)	59 (82)	0.212	0.494	0.192
	*Outside Amsterdam*	27 (21)	11 (15)	1 (10)	8 (25)	13 (19)	23 (31)	8 (31)	8 (33)	13 (18)			
**Number of sexual partners in past 6 months** c	*Median (IQR)*	7 (3–18)	6 (3–10)	5 (3–15)	15 (10–21)	2 (2–5)	3 (2–5)	2 (1–3)	2 (1–5)	3 (2–4)	<0.001	0.001	0.656
**Notified for STI**	*No*	103 (80)	61 (76)	9 (90)	28 (82)	41 (58)	58 (75)	14 (54)	15 (60)	55 (73)	0.005	0.782	0.059
	*Yes*	25 (20)	19 (24)	1 (10)	6 (18)	30 (42)	19 (25)	12 (46)	10 (40)	20 (27)			
**STI-related complaints**	*No*	62 (48)	43 (54)	3 (30)	10 (29)	42 (59)	36 (47)	15 (58)	9 (36)	32 (43)	0.085	0.073	0.149
	*Yes*	66 (52)	37 (46)	7 (70)	24 (71)	29 (41)	41 (53)	11 (42)	16 (64)	43 (57)			
**HIV** d	*Negative*	73 (59)	50 (64)	8 (80)	4 (13)	69 (97)	70 (93)	25 (100)	25 (100)	74 (99)	<0.001	<0.001	0.342
	*Positive*	50 (41)	28 (36)	2 (20)	28 (88)	2 (3)	5 (7)	0	0	1 (1)			
***Neisseria gonorrhoeae***	*Negative*	106 (83)	66 (83)	8 (80)	28 (82)	63 (89)	71 (92)	25 (96)	19 (76)	74 (99)	0.002	0.991	0.005
	*Positive*	22 (17)	14 (18)	2 (20)	6 (18)	8 (11)	6 (8)	1 (4)	6 (24)	1 (1)			

MLST: multilocus sequence typing; MSM: men who have sex with men; MSMO: men who have sex with men only; MSMW: men who have sex with men and women; MSWO: men who have sex with women only; STI: sexually transmitted infections; IQR: interquartile range.

p_total_ represents the p-value for the analyses over all clusters and the residual group; p_I to IV_ over MSM-associated clusters, i.e. clusters I–IV and p_V to VIII+R_ over heterosexual-associated clusters, i.e. clusters V–VIII and the residual group.

a Data were missing for 1 sample from cluster I, 1 from cluster III, 1 from cluster IV and 1 from cluster VI.

b Data were missing for 2 samples from cluster I, 5 from cluster II, 2 from cluster IV, 2 from cluster V, 3 from cluster VI, 1 from cluster VIII and 3 from the residual group.

c Data were missing for 3 samples from cluster I and 3 from cluster II.

d Data were missing for 5 samples from cluster I, 2 from cluster II, 2 from cluster IV, 2 from cluster VI and 1 from cluster VII.

Diversity within MSM-associated clusters was low. The majority of samples in clusters II, III, and IV belonged to a single sequence type, while most other sequence types differed only in 1 locus from the central sequence type (*D* = 0.38, 0.38 and 0.22, respectively; Table 2; Figure 2). Cluster I showed more variation (*D* = 0.77), as it contained 3 dominant sequence types; however, all but 1 of the remaining samples differed no more than 1 locus from these sequence types. Among heterosexuals, diversity was much higher: numerous small clusters and singletons were observed and also more variation was seen within the large heterosexual-associated clusters (*D* = 0.82, 0.86, 0.71 and 0.87, respectively for clusters V, VI, VII and VIII). Although the majority of samples belonged to 1 or 2 dominant sequence types, many more sequence type variants were present among heterosexuals and these variants could vary in up to 3 loci from the central sequence types.

### Clinical and Epidemiological Analysis

Only MSM-associated clusters II and III included some heterosexuals. Interestingly, men having sex with both men and women (MSMW) were more likely to be infected with strains from the heterosexual-associated clusters compared to men who only had sex with women (MSWO, *p* = 0.001, Figure 2).

Comparing the MSM-associated clusters, significant differences were observed in number of sexual partners (*p* = 0.001) and HIV serostatus (*p*<0.001, Table 2). When we excluded the LGV cluster (IV) from the analyses, no significant differences were found for the 3 other MSM-associated clusters.

Comparing heterosexual-associated clusters, we observed differences for ethnicity and being notified for STI by a sexual partner, but these were not significant. (Table 2). Participants infected with *C. trachomatis* types from cluster VIII were more likely to be of non-Dutch origin, whereas participants infected with *C. trachomatis* types from cluster VI were less often notified for STI by a sexual partner. We found that participants with samples in small clusters or with an infection that did not cluster were less likely to be coinfected with *N. gonorrhoeae*.

## Discussion

Using high resolution multilocus sequence typing, we identified clusters of *C. trachomatis* strains representing but not coinciding with all prevailing genovars in Amsterdam. As in previous studies, we found differences in *C. trachomatis* genovar distributions among infected MSM and heterosexuals [5], [15]. MSM were mainly infected by genovars D, G, J, and L2b, whereas heterosexuals were mainly infected by genovars D, E, F, and I. On a cluster level however, we found very distinct circulating strains for the 2 sexual risk groups. Thus MLST provided more solid proof of independent transmission and circulation, since it was genetically more discriminating than using *ompA* typing alone [11], [16], [17]. For example, 2 clusters of MSM-associated samples were identified, all of which had a genovar D *ompA* type, but these differed clearly from the heterosexual genovar D infections. The same was true for genovar G and J, showing separate clusters for MSM and heterosexuals. Differences in genovar distributions between the 2 sexual risk groups were described previously [2]–[7]. This is also in concordance with the findings of *Christerson et al*. (2012), describing differences in the distribution of *C. trachomatis* strains between MSM and women in both Sweden and the Netherlands [12]. Therefore, we think that distinct transmission of *C. trachomatis* strains is not unique for Amsterdam.

Recent full genome studies revealed that *ompA* is more mobile, due to recombination, than previously thought [18]. Using 5 extra genomic targets next to *ompA*, we were able to show that samples with identical *ompA* types can belong to different clusters, even if these clusters are associated with the same risk groups, such as the genovar D clusters among MSM and the genovar E clusters among heterosexuals (Figure 1, Figure 2, Table 2, Table S1). The opposite also occurs as different genovars can clearly belong to the same cluster. Among MSM, a cluster was indentified, showing a probable recombination event between a genovar G and a genovar J type. Among heterosexuals, we identified a cluster being largely of a genovar F type, but which recombined with a genovar J type *ompA* and two distinct genovar D types. High resolution MLST is able to discern close relationships in spite of recombination events, while *ompA* typing does not. Therefore it is a very useful tool for epidemiological studies. Previously described MLST schemes use housekeeping genes and canonical SNPs since they seek to answer evolutionary questions, such as establishing *Chlamydia* relationships at the genus level [19], [20]. Our modified MLST scheme however, makes use of genes that are under immune pressure or have variable repeat regions. This enables to demonstrate detailed genetic differences between *C. trachomatis* strains that are involved in transmission chains in human hosts in a short time frame of only a few years.

The thus identified genetic diversity of the MSM-associated chlamydial population was very low, compared to that of the heterosexual-associated strains. Nearly all samples from MSM were found in just 4 homogeneous clusters, pointing to clonal outbreaks. The variation in types found among heterosexuals was much larger. Not only were the large heterosexual-associated clusters more heterogeneous, there were also numerous small clusters and singletons. These differences might be explained by host-related factors, such as differences in transmission networks and population size, as well as by pathogen-related factors, such as differences in tissue tropism.

Clear epidemiological differences between *C. trachomatis*-infected MSM and heterosexuals were observed already at intake in Amsterdam. Infected MSM were often in their late thirties and reported a median of 8 sexual partners in the past 6 months, whereas infected heterosexuals were mostly in their early twenties and reported just 1 or 2 partners. We found that exchange of strains between MSM and heterosexuals was rare, which was supported by the inclusion of only 8 bisexual men in our study. The statistical analyses of MSM-associated clusters revealed that a subpopulation existed, consisting primarily of HIV-infected men with higher sexual risk behavior, in which LGV circulated [10]. Men in the 3 non-LGV MSM-associated clusters however did not differ in their demographic or sexual behavior characteristics. This suggests that the non-LGV *C. trachomatis* strains were not circulating in separate subpopulations of MSM in Amsterdam. The existence of a broad behavioral transmission network may well explain the lower diversity within chlamydial strains found among MSM. Differences in being notified for STI and in ethnicity between clusters associated with heterosexuals suggest the existence of subpopulations among heterosexuals. Together with the lower number of partners for heterosexuals, this may have led to the higher genetic diversity of the chlamydial strains. Also for other infections a clear separation of pathogen types has been reported for MSM versus heterosexuals, notably for all hepatitis viruses (HAV, HBV, and HCV) and *N. gonorrhoeae* [21]–[25].

Recently, an alternative explanation for the different distributions of genotypes has been put forward. *Jeffrey et al*. (2010) suggested that this difference could be explained by pathogen-related factors, such as tissue tropism [26]. For LGV, we found a clear preference for proctal tissue, as LGV was detected in only one urine sample in this study and all other LGV-infections were found in proctal samples. Strains from the other MSM-associated clusters clearly showed the ability to infect urethral tissue, but these strains may have adapted to favor infection of proctal tissue over cervical tissue. This could explain why only one sample coming from a woman was infected with an MSM-associated strain. We do not know whether MSM-associated strains can be found in proctal samples from women, as no such samples were available for the present study.

A weakness in our study is that the participants of this study were recruited in 2 consecutive time periods, MSM in the first and heterosexuals in the second period, but together a rather short period of less than 24 months. This might have influenced cluster formation, but previous studies demonstrated that the mutation rate of the *C. trachomatis* genome is very low [26]. In addition, sampling among MSM might have been biased towards urethral types. As only 11 proctal samples were excluded due to the presence of a concurrent urine sample, we assume this effect is minimal.

Another limitation is that all participants were recruited at the STI outpatient clinic, which may have introduced bias towards higher risk behavior and may not be representative for the general *C. trachomatis*-infected population. This applies to MSM in Amsterdam and even more to heterosexuals. In addition, many patients with asymptomatic infections and those who visited their general practitioner were obviously not included in the present study.

Despite these limitations, we are confident that the distinct *C. trachomatis* transmission patterns among MSM and heterosexuals in Amsterdam are true and reliable. Our typing data show a more clonal character for strains circulating among MSM and ongoing diversifying transmission of strains circulating among heterosexuals. We do not know presently whether this is a global or a local phenomenon, although similar findings have been found for sets of samples from Sweden, the Netherlands and the United States [12]. These findings on *C. trachomatis* distribution and transmission patterns therefore need to be confirmed in a more global setting by MLST typing and cluster analysis of samples from both MSM and heterosexuals in countries where *C. trachomatis* infection is highly prevalent. This would contribute to a large worldwide database (mlstdb.bmc.uu.se), and would improve insight into the global variation found within and between *C. trachomatis* strains. Finally, the effect of mixing patterns of the hosts versus pathogen-related factors on the distribution of chlamydial strains needs to be clarified. This knowledge will contribute to a better understanding of chlamydial transmission and help to improve screening and prevention programs.

## Supporting Information

Table S1 **MLST-data of the 526 samples collected at the STI outpatient clinic, Amsterdam, July 2008–May 2010.** Coding is according to the *Chlamydia trachomatis* MLST database (mlstdb.bmc.uu.se). The samples are sorted by cluster and sequence type.
(DOC)
