# Supplementary material for: Distinct Transmission Networks of Chlamydia trachomatis in Men Who Have Sex with Men and Heterosexual Adults in Amsterdam, The Netherlands
Source: PLoS One. 2013 Jan 16;8(1):e53869. doi: 10.1371/journal.pone.0053869 (PMC3547048; doi:10.1371/journal.pone.0053869)
Supplement: Table S1 — MLST-data of the 526 samples collected at the STI outpatient clinic, Amsterdam, July 2008–May 2010. Coding is according to the Chlamydia trachomatis MLST database (mlstdb.bmc.uu.se). The samples are sorted by cluster and sequence type. (DOC) [file pone.0053869.s001.doc]

*Supplementary Table 1. MLST-data of the 526 samples collected at the STI outpatient clinic, Amsterdam, July 2008–May 2010. Coding is according to the Chlamydia trachomatis MLST database (mlstdb.bmc.uu.se). The samples are sorted by cluster and sequence type*.

| **Cluster name** | **Sample name** | **MLST-6 ST** | **genovar** | ***ompA* (CT681)** | ***hctB* (CT046)** | **CT058** | **CT144** | **CT172** | ***pbpB* (CT682)** |
| --- | --- | --- | --- | --- | --- | --- | --- | --- | --- |
| **Cluster I** | 2005 | 33 | G | 8 | 10 | 8 | 5 | 3 | 6 |
|  | 2006 | 33 | G | 8 | 10 | 8 | 5 | 3 | 6 |
|  | 2009 | 33 | G | 8 | 10 | 8 | 5 | 3 | 6 |
|  | 2036 | 33 | G | 8 | 10 | 8 | 5 | 3 | 6 |
|  | 2088 | 33 | G | 8 | 10 | 8 | 5 | 3 | 6 |
|  | 2093 | 33 | G | 8 | 10 | 8 | 5 | 3 | 6 |
|  | 2108 | 33 | G | 8 | 10 | 8 | 5 | 3 | 6 |
|  | 2117 | 33 | G | 8 | 10 | 8 | 5 | 3 | 6 |
|  | 2133 | 33 | G | 8 | 10 | 8 | 5 | 3 | 6 |
|  | 2147 | 33 | G | 8 | 10 | 8 | 5 | 3 | 6 |
|  | 2161 | 33 | G | 8 | 10 | 8 | 5 | 3 | 6 |
|  | 2166 | 33 | G | 8 | 10 | 8 | 5 | 3 | 6 |
|  | 2186 | 33 | G | 8 | 10 | 8 | 5 | 3 | 6 |
|  | 2210 | 33 | G | 8 | 10 | 8 | 5 | 3 | 6 |
|  | 2257 | 33 | G | 8 | 10 | 8 | 5 | 3 | 6 |
|  | 2263 | 33 | G | 8 | 10 | 8 | 5 | 3 | 6 |
|  | 2270 | 33 | G | 8 | 10 | 8 | 5 | 3 | 6 |
|  | 2283 | 33 | G | 8 | 10 | 8 | 5 | 3 | 6 |
|  | 2346 | 33 | G | 8 | 10 | 8 | 5 | 3 | 6 |
|  | 2349 | 33 | G | 8 | 10 | 8 | 5 | 3 | 6 |
|  | 2367 | 33 | G | 8 | 10 | 8 | 5 | 3 | 6 |
|  | 2421 | 33 | G | 8 | 10 | 8 | 5 | 3 | 6 |
|  | 2428 | 33 | G | 8 | 10 | 8 | 5 | 3 | 6 |
|  | 2004 | 52 | G | 8 | 20 | 8 | 5 | 3 | 6 |
|  | 2011 | 52 | G | 8 | 20 | 8 | 5 | 3 | 6 |
|  | 2023 | 52 | G | 8 | 20 | 8 | 5 | 3 | 6 |
|  | 2024 | 52 | G | 8 | 20 | 8 | 5 | 3 | 6 |
|  | 2029 | 52 | G | 8 | 20 | 8 | 5 | 3 | 6 |
|  | 2058 | 52 | G | 8 | 20 | 8 | 5 | 3 | 6 |
|  | 2060 | 52 | G | 8 | 20 | 8 | 5 | 3 | 6 |
|  | 2062 | 52 | G | 8 | 20 | 8 | 5 | 3 | 6 |
|  | 2068 | 52 | G | 8 | 20 | 8 | 5 | 3 | 6 |
|  | 2082 | 52 | G | 8 | 20 | 8 | 5 | 3 | 6 |
|  | 2125 | 52 | G | 8 | 20 | 8 | 5 | 3 | 6 |
|  | 2129 | 52 | G | 8 | 20 | 8 | 5 | 3 | 6 |
|  | 2130 | 52 | G | 8 | 20 | 8 | 5 | 3 | 6 |
|  | 2142 | 52 | G | 8 | 20 | 8 | 5 | 3 | 6 |
|  | 2143 | 52 | G | 8 | 20 | 8 | 5 | 3 | 6 |
|  | 2149 | 52 | G | 8 | 20 | 8 | 5 | 3 | 6 |
|  | 2154 | 52 | G | 8 | 20 | 8 | 5 | 3 | 6 |
|  | 2157 | 52 | G | 8 | 20 | 8 | 5 | 3 | 6 |
|  | 2164 | 52 | G | 8 | 20 | 8 | 5 | 3 | 6 |
|  | 2167 | 52 | G | 8 | 20 | 8 | 5 | 3 | 6 |
|  | 2171 | 52 | G | 8 | 20 | 8 | 5 | 3 | 6 |
|  | 2174 | 52 | G | 8 | 20 | 8 | 5 | 3 | 6 |
|  | 2184 | 52 | G | 8 | 20 | 8 | 5 | 3 | 6 |
|  | 2187 | 52 | G | 8 | 20 | 8 | 5 | 3 | 6 |
|  | 2193 | 52 | G | 8 | 20 | 8 | 5 | 3 | 6 |
|  | 2195 | 52 | G | 8 | 20 | 8 | 5 | 3 | 6 |
|  | 2198 | 52 | G | 8 | 20 | 8 | 5 | 3 | 6 |
|  | 2204 | 52 | G | 8 | 20 | 8 | 5 | 3 | 6 |
|  | 2219 | 52 | G | 8 | 20 | 8 | 5 | 3 | 6 |
|  | 2220 | 52 | G | 8 | 20 | 8 | 5 | 3 | 6 |
|  | 2230 | 52 | G | 8 | 20 | 8 | 5 | 3 | 6 |
|  | 2279 | 52 | G | 8 | 20 | 8 | 5 | 3 | 6 |
|  | 2294 | 52 | G | 8 | 20 | 8 | 5 | 3 | 6 |
|  | 2298 | 52 | G | 8 | 20 | 8 | 5 | 3 | 6 |
|  | 2300 | 52 | G | 8 | 20 | 8 | 5 | 3 | 6 |
|  | 2303 | 52 | G | 8 | 20 | 8 | 5 | 3 | 6 |
|  | 2304 | 52 | G | 8 | 20 | 8 | 5 | 3 | 6 |
|  | 2308 | 52 | G | 8 | 20 | 8 | 5 | 3 | 6 |
|  | 2310 | 52 | G | 8 | 20 | 8 | 5 | 3 | 6 |
|  | 2322 | 52 | G | 8 | 20 | 8 | 5 | 3 | 6 |
|  | 2329 | 52 | G | 8 | 20 | 8 | 5 | 3 | 6 |
|  | 2333 | 52 | G | 8 | 20 | 8 | 5 | 3 | 6 |
|  | 2338 | 52 | G | 8 | 20 | 8 | 5 | 3 | 6 |
|  | 2369 | 52 | G | 8 | 20 | 8 | 5 | 3 | 6 |
|  | 2429 | 52 | G | 8 | 20 | 8 | 5 | 3 | 6 |
|  | 2435 | 52 | G | 8 | 20 | 8 | 5 | 3 | 6 |
|  | 2436 | 52 | G | 8 | 20 | 8 | 5 | 3 | 6 |
|  | 2437 | 52 | G | 8 | 20 | 8 | 5 | 3 | 6 |
|  | 2442 | 52 | G | 8 | 20 | 8 | 5 | 3 | 6 |
|  | 2256 | 108a | G | 8 | 29 | 8 | 5 | 3 | 6 |
|  | 2261 | 108a | G | 8 | 29 | 8 | 5 | 3 | 6 |
|  | 2272 | 108a | G | 8 | 29 | 8 | 5 | 3 | 6 |
|  | 2021 | 108c | J | 53 | 29 | 8 | 5 | 3 | 6 |
|  | 2040 | 108c | J | 53 | 29 | 8 | 5 | 3 | 6 |
|  | 2044 | 108c | J | 53 | 29 | 8 | 5 | 3 | 6 |
|  | 2050 | 108c | J | 53 | 29 | 8 | 5 | 3 | 6 |
|  | 2063 | 108c | J | 53 | 29 | 8 | 5 | 3 | 6 |
|  | 2069 | 108c | J | 53 | 29 | 8 | 5 | 3 | 6 |
|  | 2074 | 108c | J | 53 | 29 | 8 | 5 | 3 | 6 |
|  | 2103 | 108c | J | 53 | 29 | 8 | 5 | 3 | 6 |
|  | 2105 | 108c | J | 53 | 29 | 8 | 5 | 3 | 6 |
|  | 2115 | 108c | J | 53 | 29 | 8 | 5 | 3 | 6 |
|  | 2131 | 108c | J | 53 | 29 | 8 | 5 | 3 | 6 |
|  | 2153 | 108c | J | 53 | 29 | 8 | 5 | 3 | 6 |
|  | 2162 | 108c | J | 53 | 29 | 8 | 5 | 3 | 6 |
|  | 2169 | 108c | J | 53 | 29 | 8 | 5 | 3 | 6 |
|  | 2182 | 108c | J | 53 | 29 | 8 | 5 | 3 | 6 |
|  | 2183 | 108c | J | 53 | 29 | 8 | 5 | 3 | 6 |
|  | 2201 | 108c | J | 53 | 29 | 8 | 5 | 3 | 6 |
|  | 2287 | 108c | J | 53 | 29 | 8 | 5 | 3 | 6 |
|  | 2309 | 108c | J | 53 | 29 | 8 | 5 | 3 | 6 |
|  | 2316 | 108c | J | 53 | 29 | 8 | 5 | 3 | 6 |
|  | 2317 | 108c | J | 53 | 29 | 8 | 5 | 3 | 6 |
|  | 2330 | 108c | J | 53 | 29 | 8 | 5 | 3 | 6 |
|  | 2348 | 108c | J | 53 | 29 | 8 | 5 | 3 | 6 |
|  | 2357 | 108c | J | 53 | 29 | 8 | 5 | 3 | 6 |
|  | 2359 | 108c | J | 53 | 29 | 8 | 5 | 3 | 6 |
|  | 2424 | 108c | J | 53 | 29 | 8 | 5 | 3 | 6 |
|  | 2427 | 108c | J | 53 | 29 | 8 | 5 | 3 | 6 |
|  | 2430 | 108c | J | 53 | 29 | 8 | 5 | 3 | 6 |
|  | 2434 | 108c | J | 53 | 29 | 8 | 5 | 3 | 6 |
|  | 2439 | 108c | J | 53 | 29 | 8 | 5 | 3 | 6 |
|  | 2025 | 112 | J | 53 | 29 | 8 | 5 | 4 | 6 |
|  | 2214 | 112 | J | 53 | 29 | 8 | 5 | 4 | 6 |
|  | 2268 | 112 | J | 53 | 29 | 8 | 5 | 4 | 6 |
|  | 2328 | 112 | J | 53 | 29 | 8 | 5 | 4 | 6 |
|  | 2365 | 112 | J | 53 | 29 | 8 | 5 | 4 | 6 |
|  | 2135 | 301 | G | 8 | 20 | 8 | 5 | 1 | 6 |
|  | 2433 | 302 | G | 8 | 20 | 8 | 5 | 3 | 4 |
|  | 2200 | 303 | G | 8 | 20 | 8 | 5 | 3 | 34 |
|  | 2106 | 304 | G | 8 | 20 | 8 | 5 | 4 | 6 |
|  | 2132 | 304 | G | 8 | 20 | 8 | 5 | 4 | 6 |
|  | 2168 | 304 | G | 8 | 20 | 8 | 5 | 4 | 6 |
|  | 2431 | 306 | G | 8 | 29 | 8 | 5 | 3 | 18 |
|  | 2354 | 307 | J | 53 | 29 | 8 | 5 | 3 | 58 |
|  | 2207 | 308 | J | 53 | 29 | 8 | 5 | 29 | 6 |
|  | 2225 | 309 | J | 53 | 29 | 8 | 17 | 3 | 6 |
|  | 2087 | 343 | J | 53 | 72 | 8 | 5 | 3 | 6 |
|  | 2079 | 344 | G | 8 | 73 | 8 | 5 | 3 | 6 |
|  | 2271 | 345 | J | 53 | 74 | 8 | 5 | 3 | 6 |
|  | 2275 | 345 | J | 53 | 74 | 8 | 5 | 3 | 6 |
|  | 2366 | 346 | G | 8 | 75 | 8 | 5 | 3 | 6 |
|  | 2075 | 352 | G | 8 | 80 | 8 | 5 | 3 | 6 |
|  | 2141 | 353 | G | 8 | 81 | 8 | 5 | 3 | 6 |
|  | 2295 | 354 | J | 53 | 82 | 8 | 5 | 3 | 6 |
| **Cluster II** | 2274 | 14 | D | 1 | 5 | 20 | 5 | 2 | 8 |
|  | 2003 | 109 | D | 1 | 5 | 20 | 5 | 2 | 34 |
|  | 2018 | 109 | D | 1 | 5 | 20 | 5 | 2 | 34 |
|  | 2032 | 109 | D | 1 | 5 | 20 | 5 | 2 | 34 |
|  | 2039 | 109 | D | 1 | 5 | 20 | 5 | 2 | 34 |
|  | 2042 | 109 | D | 1 | 5 | 20 | 5 | 2 | 34 |
|  | 2052 | 109 | D | 1 | 5 | 20 | 5 | 2 | 34 |
|  | 2054 | 109 | D | 1 | 5 | 20 | 5 | 2 | 34 |
|  | 2055 | 109 | D | 1 | 5 | 20 | 5 | 2 | 34 |
|  | 2059 | 109 | D | 1 | 5 | 20 | 5 | 2 | 34 |
|  | 2064 | 109 | D | 1 | 5 | 20 | 5 | 2 | 34 |
|  | 2067 | 109 | D | 1 | 5 | 20 | 5 | 2 | 34 |
|  | 2097 | 109 | D | 1 | 5 | 20 | 5 | 2 | 34 |
|  | 2099 | 109 | D | 1 | 5 | 20 | 5 | 2 | 34 |
|  | 2111 | 109 | D | 1 | 5 | 20 | 5 | 2 | 34 |
|  | 2113 | 109 | D | 1 | 5 | 20 | 5 | 2 | 34 |
|  | 2114 | 109 | D | 1 | 5 | 20 | 5 | 2 | 34 |
|  | 2121 | 109 | D | 1 | 5 | 20 | 5 | 2 | 34 |
|  | 2122 | 109 | D | 1 | 5 | 20 | 5 | 2 | 34 |
|  | 2124 | 109 | D | 1 | 5 | 20 | 5 | 2 | 34 |
|  | 2128 | 109 | D | 1 | 5 | 20 | 5 | 2 | 34 |
|  | 2136 | 109 | D | 1 | 5 | 20 | 5 | 2 | 34 |
|  | 2140 | 109 | D | 1 | 5 | 20 | 5 | 2 | 34 |
|  | 2148 | 109 | D | 1 | 5 | 20 | 5 | 2 | 34 |
|  | 2155 | 109 | D | 1 | 5 | 20 | 5 | 2 | 34 |
|  | 2173 | 109 | D | 1 | 5 | 20 | 5 | 2 | 34 |
|  | 2175 | 109 | D | 1 | 5 | 20 | 5 | 2 | 34 |
|  | 2178 | 109 | D | 1 | 5 | 20 | 5 | 2 | 34 |
|  | 2185 | 109 | D | 1 | 5 | 20 | 5 | 2 | 34 |
|  | 2189 | 109 | D | 1 | 5 | 20 | 5 | 2 | 34 |
|  | 2191 | 109 | D | 1 | 5 | 20 | 5 | 2 | 34 |
|  | 2196 | 109 | D | 1 | 5 | 20 | 5 | 2 | 34 |
|  | 2199 | 109 | D | 1 | 5 | 20 | 5 | 2 | 34 |
|  | 2205 | 109 | D | 1 | 5 | 20 | 5 | 2 | 34 |
|  | 2211 | 109 | D | 1 | 5 | 20 | 5 | 2 | 34 |
|  | 2212 | 109 | D | 1 | 5 | 20 | 5 | 2 | 34 |
|  | 2216 | 109 | D | 1 | 5 | 20 | 5 | 2 | 34 |
|  | 2218 | 109 | D | 1 | 5 | 20 | 5 | 2 | 34 |
|  | 2221 | 109 | D | 1 | 5 | 20 | 5 | 2 | 34 |
|  | 2222 | 109 | D | 1 | 5 | 20 | 5 | 2 | 34 |
|  | 2232 | 109 | D | 1 | 5 | 20 | 5 | 2 | 34 |
|  | 2234 | 109 | D | 1 | 5 | 20 | 5 | 2 | 34 |
|  | 2240 | 109 | D | 1 | 5 | 20 | 5 | 2 | 34 |
|  | 2247 | 109 | D | 1 | 5 | 20 | 5 | 2 | 34 |
|  | 2249 | 109 | D | 1 | 5 | 20 | 5 | 2 | 34 |
|  | 2250 | 109 | D | 1 | 5 | 20 | 5 | 2 | 34 |
|  | 2255 | 109 | D | 1 | 5 | 20 | 5 | 2 | 34 |
|  | 2259 | 109 | D | 1 | 5 | 20 | 5 | 2 | 34 |
|  | 2265 | 109 | D | 1 | 5 | 20 | 5 | 2 | 34 |
|  | 2273 | 109 | D | 1 | 5 | 20 | 5 | 2 | 34 |
|  | 2281 | 109 | D | 1 | 5 | 20 | 5 | 2 | 34 |
|  | 2284 | 109 | D | 1 | 5 | 20 | 5 | 2 | 34 |
|  | 2289 | 109 | D | 1 | 5 | 20 | 5 | 2 | 34 |
|  | 2291 | 109 | D | 1 | 5 | 20 | 5 | 2 | 34 |
|  | 2306 | 109 | D | 1 | 5 | 20 | 5 | 2 | 34 |
|  | 2326 | 109 | D | 1 | 5 | 20 | 5 | 2 | 34 |
|  | 2331 | 109 | D | 1 | 5 | 20 | 5 | 2 | 34 |
|  | 2334 | 109 | D | 1 | 5 | 20 | 5 | 2 | 34 |
|  | 2344 | 109 | D | 1 | 5 | 20 | 5 | 2 | 34 |
|  | 2396 | 109 | D | 1 | 5 | 20 | 5 | 2 | 34 |
|  | 2422 | 109 | D | 1 | 5 | 20 | 5 | 2 | 34 |
|  | 2423 | 109 | D | 1 | 5 | 20 | 5 | 2 | 34 |
|  | 2426 | 109 | D | 1 | 5 | 20 | 5 | 2 | 34 |
|  | 3274 | 109 | D | 1 | 5 | 20 | 5 | 2 | 34 |
|  | 2071 | 180 | D | 1 | 5 | 42 | 5 | 2 | 34 |
|  | 2102 | 194 | D | 1 | 5 | 6 | 5 | 2 | 34 |
|  | 2138 | 194 | D | 1 | 5 | 6 | 5 | 2 | 34 |
|  | 2296 | 194 | D | 1 | 5 | 6 | 5 | 2 | 34 |
|  | 2243 | 207 | D | 1 | 5 | 45 | 5 | 2 | 34 |
|  | 3256 | 242 | D | 1 | 5 | 20 | 5 | 2 | 2 |
|  | 2278 | 244 | D | 1 | 5 | 20 | 5 | 2 | 55 |
|  | 2030 | 245 | D | 1 | 5 | 20 | 5 | 19 | 34 |
|  | 2095 | 246 | D | 1 | 5 | 20 | 5 | 21 | 34 |
|  | 3238 | 247 | D | 1 | 5 | 20 | 36 | 2 | 34 |
|  | 2116 | 311 | D | 1 | 29 | 20 | 5 | 2 | 34 |
|  | 2083 | 318 | D | 1 | 45 | 20 | 5 | 2 | 34 |
|  | 2019 | 324 | D | 1 | 55 | 20 | 5 | 2 | 34 |
|  | 2026 | 324 | D | 1 | 55 | 20 | 5 | 2 | 34 |
|  | 2038 | 324 | D | 1 | 55 | 20 | 5 | 2 | 34 |
|  | 2282 | 324 | D | 1 | 55 | 20 | 5 | 2 | 34 |
| **Cluster III** | 2031 | 11 | D | 1 | 5 | 19 | 7 | 2 | 10 |
|  | 2160 | 11 | D | 1 | 5 | 19 | 7 | 2 | 10 |
|  | 2209 | 11 | D | 1 | 5 | 19 | 7 | 2 | 10 |
|  | 2241 | 11 | D | 1 | 5 | 19 | 7 | 2 | 10 |
|  | 2242 | 11 | D | 1 | 5 | 19 | 7 | 2 | 10 |
|  | 2286 | 11 | D | 1 | 5 | 19 | 7 | 2 | 10 |
|  | 2355 | 11 | D | 1 | 5 | 19 | 7 | 2 | 10 |
|  | 2368 | 11 | D | 1 | 5 | 19 | 7 | 2 | 10 |
|  | 2327 | 195 | D | 1 | 45 | 19 | 7 | 2 | 10 |
|  | 3278 | 312 | D | 1 | 35 | 19 | 7 | 2 | 10 |
| **Cluster IV** | 2288 | 58a | L2 | 22 | 27 | 13 | 17 | 13 | 28 |
|  | 2301 | 58a | L2 | 22 | 27 | 13 | 17 | 13 | 28 |
|  | 2339 | 58a | L2 | 22 | 27 | 13 | 17 | 13 | 28 |
|  | 2179 | 58b | L2b | 28 | 27 | 13 | 17 | 13 | 28 |
|  | 2194 | 58b | L2b | 28 | 27 | 13 | 17 | 13 | 28 |
|  | 2202 | 58b | L2b | 28 | 27 | 13 | 17 | 13 | 28 |
|  | 2203 | 58b | L2b | 28 | 27 | 13 | 17 | 13 | 28 |
|  | 2290 | 58b | L2b | 28 | 27 | 13 | 17 | 13 | 28 |
|  | 2297 | 58b | L2b | 28 | 27 | 13 | 17 | 13 | 28 |
|  | 2318 | 58b | L2b | 28 | 27 | 13 | 17 | 13 | 28 |
|  | 2341 | 58b | L2b | 28 | 27 | 13 | 17 | 13 | 28 |
|  | 2370 | 58b | L2b | 28 | 27 | 13 | 17 | 13 | 28 |
|  | 2371 | 58b | L2b | 28 | 27 | 13 | 17 | 13 | 28 |
|  | 2372 | 58b | L2b | 28 | 27 | 13 | 17 | 13 | 28 |
|  | 2373 | 58b | L2b | 28 | 27 | 13 | 17 | 13 | 28 |
|  | 2375 | 58b | L2b | 28 | 27 | 13 | 17 | 13 | 28 |
|  | 2376 | 58b | L2b | 28 | 27 | 13 | 17 | 13 | 28 |
|  | 2377 | 58b | L2b | 28 | 27 | 13 | 17 | 13 | 28 |
|  | 2378 | 58b | L2b | 28 | 27 | 13 | 17 | 13 | 28 |
|  | 2379 | 58b | L2b | 28 | 27 | 13 | 17 | 13 | 28 |
|  | 2380 | 58b | L2b | 28 | 27 | 13 | 17 | 13 | 28 |
|  | 2382 | 58b | L2b | 28 | 27 | 13 | 17 | 13 | 28 |
|  | 2383 | 58b | L2b | 28 | 27 | 13 | 17 | 13 | 28 |
|  | 2384 | 58b | L2b | 28 | 27 | 13 | 17 | 13 | 28 |
|  | 2385 | 58b | L2b | 28 | 27 | 13 | 17 | 13 | 28 |
|  | 2387 | 58b | L2b | 28 | 27 | 13 | 17 | 13 | 28 |
|  | 2389 | 58b | L2b | 28 | 27 | 13 | 17 | 13 | 28 |
|  | 2390 | 58b | L2b | 28 | 27 | 13 | 17 | 13 | 28 |
|  | 2391 | 58b | L2b | 28 | 27 | 13 | 17 | 13 | 28 |
|  | 2392 | 58b | L2b | 28 | 27 | 13 | 17 | 13 | 28 |
|  | 2393 | 58b | L2b | 28 | 27 | 13 | 17 | 13 | 28 |
|  | 2394 | 58b | L2b | 28 | 27 | 13 | 17 | 13 | 28 |
|  | 2404 | 58b | L2b | 28 | 27 | 13 | 17 | 13 | 28 |
|  | 2190 | 143 | L2b | 28 | 44 | 13 | 17 | 13 | 28 |
| **Cluster V** | 3046 | 12a | D | 1 | 5 | 19 | 7 | 1 | 4 |
|  | 3100 | 12a | D | 1 | 5 | 19 | 7 | 1 | 4 |
|  | 3259 | 12a | D | 1 | 5 | 19 | 7 | 1 | 4 |
|  | 3268 | 12a | D | 1 | 5 | 19 | 7 | 1 | 4 |
|  | 3185 | 12b | D | 2 | 5 | 19 | 7 | 1 | 4 |
|  | 2101 | 12d | F | 24 | 5 | 19 | 7 | 1 | 4 |
|  | 3004 | 12d | F | 24 | 5 | 19 | 7 | 1 | 4 |
|  | 3005 | 12d | F | 24 | 5 | 19 | 7 | 1 | 4 |
|  | 3030 | 12d | F | 24 | 5 | 19 | 7 | 1 | 4 |
|  | 3032 | 12d | F | 24 | 5 | 19 | 7 | 1 | 4 |
|  | 3052 | 12d | F | 24 | 5 | 19 | 7 | 1 | 4 |
|  | 3055 | 12d | F | 24 | 5 | 19 | 7 | 1 | 4 |
|  | 3060 | 12d | F | 24 | 5 | 19 | 7 | 1 | 4 |
|  | 3062 | 12d | F | 24 | 5 | 19 | 7 | 1 | 4 |
|  | 3065 | 12d | F | 24 | 5 | 19 | 7 | 1 | 4 |
|  | 3067 | 12d | F | 24 | 5 | 19 | 7 | 1 | 4 |
|  | 3083 | 12d | F | 24 | 5 | 19 | 7 | 1 | 4 |
|  | 3089 | 12d | F | 24 | 5 | 19 | 7 | 1 | 4 |
|  | 3094 | 12d | F | 24 | 5 | 19 | 7 | 1 | 4 |
|  | 3102 | 12d | F | 24 | 5 | 19 | 7 | 1 | 4 |
|  | 3103 | 12d | F | 24 | 5 | 19 | 7 | 1 | 4 |
|  | 3117 | 12d | F | 24 | 5 | 19 | 7 | 1 | 4 |
|  | 3128 | 12d | F | 24 | 5 | 19 | 7 | 1 | 4 |
|  | 3153 | 12d | F | 24 | 5 | 19 | 7 | 1 | 4 |
|  | 3216 | 12d | F | 24 | 5 | 19 | 7 | 1 | 4 |
|  | 3222 | 12d | F | 24 | 5 | 19 | 7 | 1 | 4 |
|  | 3234 | 12d | F | 24 | 5 | 19 | 7 | 1 | 4 |
|  | 3241 | 12d | F | 24 | 5 | 19 | 7 | 1 | 4 |
|  | 3257 | 12d | F | 24 | 5 | 19 | 7 | 1 | 4 |
|  | 3263 | 12d | F | 24 | 5 | 19 | 7 | 1 | 4 |
|  | 3292 | 12d | F | 24 | 5 | 19 | 7 | 1 | 4 |
|  | 3295 | 12d | F | 24 | 5 | 19 | 7 | 1 | 4 |
|  | 3325 | 12d | F | 24 | 5 | 19 | 7 | 1 | 4 |
|  | 3327 | 12d | F | 24 | 5 | 19 | 7 | 1 | 4 |
|  | 3066 | 12e | J | 38 | 5 | 19 | 7 | 1 | 4 |
|  | 3284 | 12e | J | 38 | 5 | 19 | 7 | 1 | 4 |
|  | 3017 | 13a | D | 2 | 5 | 19 | 15 | 1 | 4 |
|  | 2441 | 13b | F | 24 | 5 | 19 | 15 | 1 | 4 |
|  | 3313 | 62a | D | 1 | 5 | 19 | 7 | 3 | 4 |
|  | 3314 | 62a | D | 1 | 5 | 19 | 7 | 3 | 4 |
|  | 3220 | 62b | F | 24 | 5 | 19 | 7 | 3 | 4 |
|  | 2236 | 90 | F | 24 | 5 | 19 | 1 | 1 | 4 |
|  | 3018 | 90 | F | 24 | 5 | 19 | 1 | 1 | 4 |
|  | 3159 | 90 | F | 24 | 5 | 19 | 1 | 1 | 4 |
|  | 3189 | 90 | F | 24 | 5 | 19 | 1 | 1 | 4 |
|  | 3285 | 90 | F | 24 | 5 | 19 | 1 | 1 | 4 |
|  | 2146 | 91 | F | 24 | 5 | 19 | 5 | 2 | 4 |
|  | 2081 | 148 | F | 24 | 5 | 19 | 7 | 2 | 4 |
|  | 2150 | 148 | F | 24 | 5 | 19 | 7 | 2 | 4 |
|  | 2350 | 148 | F | 24 | 5 | 19 | 7 | 2 | 4 |
|  | 3053 | 148 | F | 24 | 5 | 19 | 7 | 2 | 4 |
|  | 3073 | 148 | F | 24 | 5 | 19 | 7 | 2 | 4 |
|  | 3079 | 148 | F | 24 | 5 | 19 | 7 | 2 | 4 |
|  | 2158 | 179 | F | 24 | 5 | 2 | 7 | 2 | 4 |
|  | 2438 | 224 | F | 24 | 1 | 19 | 5 | 2 | 4 |
|  | 3270 | 231 | F | 24 | 5 | 19 | 1 | 2 | 4 |
|  | 3039 | 233 | J | 38 | 5 | 19 | 5 | 1 | 4 |
|  | 3020 | 235 | D | 1 | 5 | 19 | 7 | 1 | 59 |
|  | 3283 | 238a | D | 1 | 5 | 19 | 7 | 15 | 4 |
|  | 3279 | 238b | F | 24 | 5 | 19 | 7 | 15 | 4 |
|  | 3218 | 240 | F | 24 | 5 | 19 | 12 | 1 | 4 |
|  | 3168 | 250 | F | 24 | 5 | 51 | 7 | 3 | 4 |
|  | 3130 | 251 | F | 24 | 5 | 56 | 7 | 1 | 4 |
|  | 3007 | 297 | F | 24 | 13 | 19 | 7 | 1 | 4 |
|  | 3024 | 297 | F | 24 | 13 | 19 | 7 | 1 | 4 |
|  | 3120 | 332 | F | 24 | 62 | 19 | 7 | 1 | 4 |
|  | 3239 | 333 | F | 24 | 63 | 19 | 5 | 2 | 4 |
|  | 3101 | 337 | F | 24 | 66 | 19 | 7 | 1 | 4 |
|  | 3108 | 337 | F | 24 | 66 | 19 | 7 | 1 | 4 |
|  | 3111 | 337 | F | 24 | 66 | 19 | 7 | 1 | 4 |
|  | 3169 | 337 | F | 24 | 66 | 19 | 7 | 1 | 4 |
| **Cluster VI** | 3021 | 16 | E | 6 | 7 | 19 | 14 | 2 | 1 |
|  | 3049 | 16 | E | 6 | 7 | 19 | 14 | 2 | 1 |
|  | 3115 | 16 | E | 6 | 7 | 19 | 14 | 2 | 1 |
|  | 3145 | 16 | E | 6 | 7 | 19 | 14 | 2 | 1 |
|  | 3155 | 16 | E | 6 | 7 | 19 | 14 | 2 | 1 |
|  | 3286 | 16 | E | 6 | 7 | 19 | 14 | 2 | 1 |
|  | 2027 | 56a | E | 6 | 1 | 19 | 7 | 2 | 1 |
|  | 2094 | 56a | E | 6 | 1 | 19 | 7 | 2 | 1 |
|  | 2165 | 56a | E | 6 | 1 | 19 | 7 | 2 | 1 |
|  | 2228 | 56a | E | 6 | 1 | 19 | 7 | 2 | 1 |
|  | 2248 | 56a | E | 6 | 1 | 19 | 7 | 2 | 1 |
|  | 3009 | 56a | E | 6 | 1 | 19 | 7 | 2 | 1 |
|  | 3023 | 56a | E | 6 | 1 | 19 | 7 | 2 | 1 |
|  | 3033 | 56a | E | 6 | 1 | 19 | 7 | 2 | 1 |
|  | 3037 | 56a | E | 6 | 1 | 19 | 7 | 2 | 1 |
|  | 3043 | 56a | E | 6 | 1 | 19 | 7 | 2 | 1 |
|  | 3071 | 56a | E | 6 | 1 | 19 | 7 | 2 | 1 |
|  | 3076 | 56a | E | 6 | 1 | 19 | 7 | 2 | 1 |
|  | 3084 | 56a | E | 6 | 1 | 19 | 7 | 2 | 1 |
|  | 3126 | 56a | E | 6 | 1 | 19 | 7 | 2 | 1 |
|  | 3139 | 56a | E | 6 | 1 | 19 | 7 | 2 | 1 |
|  | 3147 | 56a | E | 6 | 1 | 19 | 7 | 2 | 1 |
|  | 3160 | 56a | E | 6 | 1 | 19 | 7 | 2 | 1 |
|  | 3172 | 56a | E | 6 | 1 | 19 | 7 | 2 | 1 |
|  | 3200 | 56a | E | 6 | 1 | 19 | 7 | 2 | 1 |
|  | 3230 | 56a | E | 6 | 1 | 19 | 7 | 2 | 1 |
|  | 3246 | 56a | E | 6 | 1 | 19 | 7 | 2 | 1 |
|  | 3261 | 56a | E | 6 | 1 | 19 | 7 | 2 | 1 |
|  | 3269 | 56a | E | 6 | 1 | 19 | 7 | 2 | 1 |
|  | 3321 | 56a | E | 6 | 1 | 19 | 7 | 2 | 1 |
|  | 3191 | 56b | E | 58 | 1 | 19 | 7 | 2 | 1 |
|  | 3059 | 56c | E | 61 | 1 | 19 | 7 | 2 | 1 |
|  | 2014 | 59 | E | 6 | 7 | 19 | 7 | 2 | 1 |
|  | 2151 | 59 | E | 6 | 7 | 19 | 7 | 2 | 1 |
|  | 3013 | 59 | E | 6 | 7 | 19 | 7 | 2 | 1 |
|  | 3057 | 59 | E | 6 | 7 | 19 | 7 | 2 | 1 |
|  | 3058 | 59 | E | 6 | 7 | 19 | 7 | 2 | 1 |
|  | 3063 | 59 | E | 6 | 7 | 19 | 7 | 2 | 1 |
|  | 3075 | 59 | E | 6 | 7 | 19 | 7 | 2 | 1 |
|  | 3081 | 59 | E | 6 | 7 | 19 | 7 | 2 | 1 |
|  | 3106 | 59 | E | 6 | 7 | 19 | 7 | 2 | 1 |
|  | 3113 | 59 | E | 6 | 7 | 19 | 7 | 2 | 1 |
|  | 3133 | 59 | E | 6 | 7 | 19 | 7 | 2 | 1 |
|  | 3148 | 59 | E | 6 | 7 | 19 | 7 | 2 | 1 |
|  | 3175 | 59 | E | 6 | 7 | 19 | 7 | 2 | 1 |
|  | 3229 | 59 | E | 6 | 7 | 19 | 7 | 2 | 1 |
|  | 3262 | 64a | E | 6 | 4 | 19 | 7 | 2 | 1 |
|  | 3070 | 69 | E | 6 | 5 | 19 | 6 | 2 | 2 |
|  | 3072 | 69 | E | 6 | 5 | 19 | 6 | 2 | 2 |
|  | 3176 | 69 | E | 6 | 5 | 19 | 6 | 2 | 2 |
|  | 3303 | 69 | E | 6 | 5 | 19 | 6 | 2 | 2 |
|  | 3011 | 153 | E | 6 | 35 | 19 | 7 | 2 | 1 |
|  | 3025 | 153 | E | 6 | 35 | 19 | 7 | 2 | 1 |
|  | 3105 | 153 | E | 6 | 35 | 19 | 7 | 2 | 1 |
|  | 3125 | 153 | E | 6 | 35 | 19 | 7 | 2 | 1 |
|  | 3131 | 154 | E | 6 | 48 | 19 | 7 | 2 | 1 |
|  | 3192 | 157 | E | 6 | 5 | 19 | 7 | 1 | 1 |
|  | 2096 | 171 | E | 6 | 49 | 19 | 7 | 2 | 1 |
|  | 3003 | 171 | E | 6 | 49 | 19 | 7 | 2 | 1 |
|  | 3196 | 171 | E | 6 | 49 | 19 | 7 | 2 | 1 |
|  | 3247 | 174 | E | 6 | 5 | 19 | 7 | 1 | 2 |
|  | 3248 | 174 | E | 6 | 5 | 19 | 7 | 1 | 2 |
|  | 3276 | 174 | E | 6 | 5 | 19 | 7 | 1 | 2 |
|  | 3221 | 225 | E | 6 | 1 | 19 | 6 | 2 | 1 |
|  | 3251 | 226 | E | 6 | 1 | 19 | 7 | 2 | 61 |
|  | 2070 | 236 | E | 6 | 5 | 19 | 7 | 2 | 1 |
|  | 3232 | 236 | E | 6 | 5 | 19 | 7 | 2 | 1 |
|  | 3061 | 237 | E | 6 | 5 | 19 | 7 | 2 | 2 |
|  | 3207 | 252 | E | 6 | 7 | 19 | 7 | 1 | 1 |
|  | 3197 | 255 | E | 6 | 7 | 52 | 7 | 2 | 1 |
|  | 2109 | 310 | E | 6 | 29 | 19 | 7 | 2 | 1 |
|  | 3134 | 313 | E | 6 | 35 | 54 | 7 | 2 | 1 |
|  | 3010 | 320 | E | 6 | 49 | 19 | 7 | 23 | 1 |
|  | 3077 | 323 | E | 6 | 54 | 19 | 7 | 2 | 1 |
|  | 3237 | 327 | E | 6 | 57 | 19 | 7 | 2 | 1 |
|  | 3288 | 327 | E | 6 | 57 | 19 | 7 | 2 | 1 |
|  | 3161 | 331 | E | 6 | 61 | 19 | 7 | 2 | 1 |
| **Cluster VII** | 3022 | 3 | E | 6 | 1 | 2 | 6 | 2 | 2 |
|  | 3027 | 3 | E | 6 | 1 | 2 | 6 | 2 | 2 |
|  | 3029 | 3 | E | 6 | 1 | 2 | 6 | 2 | 2 |
|  | 3034 | 3 | E | 6 | 1 | 2 | 6 | 2 | 2 |
|  | 3036 | 3 | E | 6 | 1 | 2 | 6 | 2 | 2 |
|  | 3078 | 3 | E | 6 | 1 | 2 | 6 | 2 | 2 |
|  | 3091 | 3 | E | 6 | 1 | 2 | 6 | 2 | 2 |
|  | 3098 | 3 | E | 6 | 1 | 2 | 6 | 2 | 2 |
|  | 3170 | 3 | E | 6 | 1 | 2 | 6 | 2 | 2 |
|  | 3173 | 3 | E | 6 | 1 | 2 | 6 | 2 | 2 |
|  | 3260 | 3 | E | 6 | 1 | 2 | 6 | 2 | 2 |
|  | 3273 | 3 | E | 6 | 1 | 2 | 6 | 2 | 2 |
|  | 3301 | 3 | E | 6 | 1 | 2 | 6 | 2 | 2 |
|  | 3328 | 3 | E | 6 | 1 | 2 | 6 | 2 | 2 |
|  | 2397 | 86 | E | 6 | 1 | 2 | 6 | 14 | 2 |
|  | 3183 | 86 | E | 6 | 1 | 2 | 6 | 14 | 2 |
|  | 3008 | 87 | E | 6 | 35 | 2 | 6 | 2 | 2 |
|  | 3164 | 172 | E | 6 | 1 | 2 | 7 | 2 | 2 |
|  | 3219 | 172 | E | 6 | 1 | 2 | 7 | 2 | 2 |
|  | 3141 | 222 | E | 6 | 1 | 5 | 6 | 2 | 2 |
|  | 3181 | 262 | E | 6 | 9 | 2 | 7 | 2 | 2 |
|  | 3184 | 305 | E | 6 | 25 | 2 | 6 | 2 | 2 |
|  | 3093 | 319 | E | 6 | 49 | 2 | 6 | 2 | 2 |
|  | 3112 | 325 | E | 6 | 56 | 2 | 6 | 2 | 2 |
|  | 3165 | 328 | E | 6 | 58 | 2 | 6 | 2 | 2 |
|  | 3310 | 342 | E | 6 | 71 | 2 | 7 | 2 | 2 |
| **Cluster VIII** | 3143 | 100b | I | 36 | 10 | 5 | 12 | 7 | 18 |
|  | 3228 | 100b | I | 36 | 10 | 5 | 12 | 7 | 18 |
|  | 3242 | 100b | I | 36 | 10 | 5 | 12 | 7 | 18 |
|  | 3329 | 100b | I | 36 | 10 | 5 | 12 | 7 | 18 |
|  | 3202 | 101 | I | 36 | 38 | 5 | 12 | 7 | 18 |
|  | 3217 | 101 | I | 36 | 38 | 5 | 12 | 7 | 18 |
|  | 3253 | 101 | I | 36 | 38 | 5 | 12 | 7 | 18 |
|  | 3302 | 101 | I | 36 | 38 | 5 | 12 | 7 | 18 |
|  | 3306 | 101 | I | 36 | 38 | 5 | 12 | 7 | 18 |
|  | 3014 | 135b | I | 36 | 10 | 5 | 12 | 4 | 18 |
|  | 3082 | 135b | I | 36 | 10 | 5 | 12 | 4 | 18 |
|  | 3099 | 135b | I | 36 | 10 | 5 | 12 | 4 | 18 |
|  | 3166 | 135b | I | 36 | 10 | 5 | 12 | 4 | 18 |
|  | 3215 | 135b | I | 36 | 10 | 5 | 12 | 4 | 18 |
|  | 3236 | 135b | I | 36 | 10 | 5 | 12 | 4 | 18 |
|  | 3309 | 135b | I | 36 | 10 | 5 | 12 | 4 | 18 |
|  | 3223 | 271 | I | 36 | 10 | 5 | 12 | 4 | 34 |
|  | 3167 | 272 | I | 36 | 10 | 5 | 12 | 24 | 18 |
|  | 3122 | 274 | I | 36 | 10 | 5 | 12 | 26 | 18 |
|  | 3104 | 276 | I | 36 | 10 | 5 | 12 | 27 | 18 |
|  | 3124 | 276 | I | 36 | 10 | 5 | 12 | 27 | 18 |
|  | 3233 | 276 | I | 36 | 10 | 5 | 12 | 27 | 18 |
|  | 3308 | 315 | I | 36 | 38 | 5 | 12 | 27 | 18 |
|  | 3123 | 316 | I | 36 | 38 | 5 | 32 | 7 | 18 |
|  | 3118 | 317 | I | 36 | 38 | 5 | 32 | 27 | 18 |
| **Residual group** | 3012 | 25b | I | 19 | 10 | 4 | 1 | 7 | 6 |
|  | 3092 | 25b | I | 19 | 10 | 4 | 1 | 7 | 6 |
|  | 3157 | 27 | G | 9 | 10 | 6 | 10 | 1 | 6 |
|  | 3244 | 27 | G | 9 | 10 | 6 | 10 | 1 | 6 |
|  | 2340 | 30 | K | 12 | 10 | 7 | 1 | 3 | 8 |
|  | 3016 | 30 | K | 12 | 10 | 7 | 1 | 3 | 8 |
|  | 3051 | 30 | K | 12 | 10 | 7 | 1 | 3 | 8 |
|  | 3319 | 30 | K | 12 | 10 | 7 | 1 | 3 | 8 |
|  | 3028 | 32 | K | 12 | 10 | 7 | 1 | 4 | 8 |
|  | 3138 | 32 | K | 12 | 10 | 7 | 1 | 4 | 8 |
|  | 3204 | 32 | K | 12 | 10 | 7 | 1 | 4 | 8 |
|  | 3300 | 32 | K | 12 | 10 | 7 | 1 | 4 | 8 |
|  | 3015 | 35 | D | 2 | 10 | 8 | 1 | 4 | 17 |
|  | 3127 | 35 | D | 2 | 10 | 8 | 1 | 4 | 17 |
|  | 3178 | 35 | D | 2 | 10 | 8 | 1 | 4 | 17 |
|  | 3199 | 35 | D | 2 | 10 | 8 | 1 | 4 | 17 |
|  | 3163 | 77 | D | 31 | 5 | 19 | 7 | 2 | 37 |
|  | 3293 | 77 | D | 31 | 5 | 19 | 7 | 2 | 37 |
|  | 3225 | 94 | G | 8 | 10 | 5 | 12 | 3 | 5 |
|  | 3299 | 99 | J | 38 | 36 | 15 | 7 | 1 | 4 |
|  | 3109 | 102 | I | 37 | 10 | 7 | 1 | 7 | 5 |
|  | 3044 | 128a | G | 8 | 10 | 8 | 1 | 4 | 5 |
|  | 3064 | 128a | G | 8 | 10 | 8 | 1 | 4 | 5 |
|  | 3041 | 130a | D | 2 | 10 | 4 | 1 | 3 | 5 |
|  | 3227 | 130b | G | 8 | 10 | 4 | 1 | 3 | 5 |
|  | 3156 | 138 | B | 30 | 11 | 7 | 12 | 3 | 18 |
|  | 3135 | 160 | E | 6 | 10 | 2 | 1 | 2 | 2 |
|  | 3080 | 165 | H | 35 | 12 | 5 | 1 | 9 | 8 |
|  | 3205 | 175a | K | 12 | 10 | 6 | 22 | 3 | 8 |
|  | 3272 | 197b | F | 24 | 1 | 2 | 7 | 2 | 6 |
|  | 3097 | 227 | E | 6 | 5 | 2 | 12 | 22 | 56 |
|  | 3085 | 232 | J | 20 | 5 | 19 | 1 | 4 | 18 |
|  | 3006 | 234 | D | 31 | 5 | 19 | 6 | 2 | 37 |
|  | 3026 | 253 | J | 20 | 7 | 19 | 7 | 2 | 18 |
|  | 3152 | 254 | E | 6 | 7 | 50 | 7 | 21 | 1 |
|  | 3203 | 256 | J | 53 | 8 | 4 | 1 | 1 | 6 |
|  | 3245 | 257 | K | 12 | 8 | 4 | 1 | 7 | 8 |
|  | 2246 | 258 | J | 20 | 8 | 5 | 12 | 3 | 18 |
|  | 3150 | 259 | B | 30 | 8 | 8 | 1 | 4 | 18 |
|  | 3096 | 263 | E | 6 | 9 | 15 | 6 | 2 | 2 |
|  | 3311 | 264 | J | 53 | 10 | 4 | 1 | 1 | 6 |
|  | 3320 | 265 | G | 10 | 10 | 4 | 1 | 3 | 7 |
|  | 3182 | 266 | D | 2 | 10 | 4 | 1 | 4 | 5 |
|  | 3110 | 267 | J | 20 | 10 | 5 | 1 | 15 | 18 |
|  | 3254 | 270 | G | 8 | 10 | 5 | 12 | 3 | 8 |
|  | 3050 | 275 | G | 8 | 10 | 5 | 12 | 27 | 5 |
|  | 3056 | 277 | G | 8 | 10 | 6 | 1 | 3 | 6 |
|  | 3045 | 279 | I | 37 | 10 | 7 | 1 | 4 | 5 |
|  | 3307 | 279 | I | 37 | 10 | 7 | 1 | 4 | 5 |
|  | 3324 | 281a | J | 20 | 10 | 8 | 1 | 4 | 18 |
|  | 3289 | 283 | D | 2 | 10 | 8 | 10 | 1 | 17 |
|  | 3252 | 286 | D | 62 | 10 | 8 | 22 | 3 | 17 |
|  | 3266 | 286 | D | 62 | 10 | 8 | 22 | 3 | 17 |
|  | 3069 | 287 | G | 8 | 10 | 8 | 22 | 7 | 6 |
|  | 3088 | 288 | G | 8 | 10 | 8 | 22 | 7 | 57 |
|  | 3119 | 288 | G | 8 | 10 | 8 | 22 | 7 | 57 |
|  | 3162 | 288 | G | 8 | 10 | 8 | 22 | 7 | 57 |
|  | 3208 | 289 | D | 1 | 10 | 8 | 34 | 1 | 4 |
|  | 3054 | 290 | I | 37 | 10 | 46 | 1 | 7 | 5 |
|  | 3179 | 294 | H | 35 | 12 | 5 | 1 | 17 | 8 |
|  | 3194 | 295 | J | 20 | 12 | 5 | 2 | 2 | 18 |
|  | 3224 | 295 | J | 20 | 12 | 5 | 2 | 2 | 18 |
|  | 3264 | 296 | G | 8 | 12 | 5 | 11 | 9 | 5 |
|  | 3271 | 296 | G | 8 | 12 | 5 | 11 | 9 | 5 |
|  | 3146 | 298 | D | 1 | 13 | 19 | 7 | 3 | 10 |
|  | 3304 | 299 | F | 24 | 13 | 19 | 12 | 2 | 4 |
|  | 3186 | 300 | F | 24 | 14 | 4 | 5 | 1 | 4 |
|  | 3177 | 321 | E | 6 | 50 | 19 | 1 | 2 | 2 |
|  | 3312 | 326 | E | 60 | 57 | 2 | 6 | 2 | 2 |
|  | 3277 | 338 | E | 6 | 67 | 2 | 7 | 2 | 60 |
|  | 3281 | 338 | E | 6 | 67 | 2 | 7 | 2 | 60 |
|  | 3198 | 340 | K | 12 | 69 | 53 | 22 | 3 | 8 |
|  | 3095 | 341 | E | 6 | 70 | 57 | 7 | 2 | 1 |
|  | 3297 | 348 | J | 20 | 77 | 5 | 12 | 4 | 18 |
|  | 3090 | 355 | D | 2 | 83 | 4 | 1 | 4 | 17 |

*MLST: multilocus sequence typing; ST: sequence type.*

*MLST-6 ST is based on ompA, CT046, CT058, CT144, CT172, and CT682; genovar is based on ompA.*
